# Wearable Ball-Impact Piezoelectric Multi-Converters for Low-Frequency Energy Harvesting from Human Motion

**DOI:** 10.3390/s22030772

**Published:** 2022-01-20

**Authors:** Alessandro Nastro, Nicola Pienazza, Marco Baù, Pietro Aceti, Markku Rouvala, Raffaele Ardito, Marco Ferrari, Alberto Corigliano, Vittorio Ferrari

**Affiliations:** 1 Department of Information Engineering, University of Brescia, 25123 Brescia, Italy; nicola.pienazza@unibs.it (N.P.); marco.bau@unibs.it (M.B.); marco.ferrari@unibs.it (M.F.); vittorio.ferrari@unibs.it (V.F.); 2 Department of Civil and Environmental Engineering, Politecnico di Milano, 20133 Milano, Italy; pietro.aceti@polimi.it (P.A.); raffaele.ardito@polimi.it (R.A.); alberto.corigliano@polimi.it (A.C.); 3 Huawei Technologies Oy, 00180 Helsinki, Finland; mrouvala@gmail.com

**Keywords:** energy harvesting, piezoelectric converter, wearable, wrist-worn, multi-degree-of-freedom converters, mono-axial harvester, bi-axial harvester, ball-impact, frequency-up conversion technique

## Abstract

Multi-converter piezoelectric harvesters based on mono-axial and bi-axial configurations are proposed. The harvesters exploit two and four piezoelectric converters (PCs) and adopt an impinging spherical steel ball to harvest electrical energy from human motion. When the harvester undergoes a shake, a tilt, or a combination of the two, the ball hits one PC, inducing an impact-based frequency-up conversion. Prototypes of the harvesters have been designed, fabricated, fastened to the wrist of a person by means of a wristband and watchband, and experimentally tested for different motion levels. The PCs of the harvesters have been fed to passive diode-based voltage-doubler rectifiers connected in parallel to a storage capacitor, *C*_s_ = 220 nF. By employing the mono-axial harvester, after 8.5 s of consecutive impacts induced by rotations of the wrist, a voltage *v*_cs_(t) of 40.2 V across the capacitor was obtained, which corresponded to a stored energy of 178 μJ. By employing the bi-axial harvester, the peak instantaneous power provided by the PCs to an optimal resistive load was 1.58 mW, with an average power of 9.65 μW over 0.7 s. The proposed harvesters are suitable to scavenge electrical energy from low-frequency nonperiodical mechanical movements, such as human motion.

## 1. Introduction

The development and employment of wearable electronic devices have rapidly increased in recent years. Their global market size has been valued at USD 32.63 billion in 2019 and is projected to further increase in the next years [1]. The interest in wearable electronic devices is due to the considerably wide number of possible applications, ranging from biomedical [2,3,4,5] to robotics [6,7] and from consumer [8,9,10] to industrial [11,12]. Wearable electronics have to fulfil some basic requirements, such as biocompatibility, light weight, and, in particular, low-energy consumption [13]. Thanks to the continuous development of electronic components that require less and less power [14,15], the possibility to harvest electrical energy from the environment is becoming both of interest and potentially viable. The effectiveness of the energy harvesting techniques has been proven by the development of battery-less autonomous sensor modules [16,17,18,19].

Furthermore, depending on the available environmental energy forms, different harvesting principles have been employed, including electro-magnetic [20,21,22], photovoltaic [23], triboelectric [24], pyroelectric [25] and piezoelectric [26,27,28,29,30] principles.

Specifically, piezoelectric converters have been extensively investigated in energy harvesting systems for the conversion of mechanical energy produced by human movements into electrical energy useful to power wearable electronics devices [31,32,33,34]. When operating in linear regime as resonant converters, piezoelectric energy harvesters have the best effectiveness if the input vibration frequencies are close to the resonant frequency of the converter [35,36,37].

However, as a consequence of the desirable reduction of harvester dimensions, the resonant frequency typically becomes higher than the frequency of the input vibrations. Furthermore, typical human movements often have a nonperiodical or random behavior over a frequency bandwidth, typically not exceeding a few hertz [38], thus limiting the practical adoption of linear energy harvesters of acceptable size. To overcome these frequency mismatches, different approaches have been developed, such as the adoption of eccentric weight [39], nonlinear mechanisms [40,41,42], or the use of impact-based and frequency-up conversion techniques [43,44,45,46]. An energy harvester system with eight nonlinear lead zirconate titanate (PZT) buckled bridges has been employed to power a commercial tire pressure monitoring systems in real time [47]. The reported system has been tested with a gear-induced interwell oscillation mechanism. Experimental results showed that an output power of 8.9 mW across the optimal resistance of 3 kΩ at 8.3 Hz rotational frequency was achieved.

Typically, impact-based frequency-up conversion techniques employ a driving structure that is sensitive to the input excitation along only one spatial direction, which repeatedly hits a piezoelectric converter [48]. An improvement can be achieved by employing a rigid ball, confined within a predefined volume, as the moving element impacting a piezoelectric converter, thus leading to an energy harvesting mechanism that is sensitive to input excitations along multiple spatial directions. An impact-based energy harvester that employs a single sodium potassium niobate (KNN) piezoelectric cantilever with a trapezoidal shape has been reported [49]. Experimental results show that, considering a ball with a radius of 500 μm, the maximum harvested power is 44 nW across a resistor of 7.3 kΩ with an applied vibration acceleration level of 4 g at a frequency of 190 Hz. Alternatively, a pendulum ball impact-excited piezoelectric energy harvester has been reported [50]. Experimental results show that for a pendulum ball excited with a 2.5 mm horizontal displacement at 2 Hz, the open-circuit peak voltage is 15.8 V and a maximum output power of 10.53 μW is achieved across a 130 kΩ load resistance.

In this context, the present work proposes wearable mono-axial and bi-axial ball-impact multi-converter piezoelectric harvesters to scavenge energy from human motion. The proposed harvesters exploit multiple piezoelectric converters configured as cantilevers which form the boundary region for the path of a steel ball. The ball-impact harvesters use the driving ball to obtain a compact structure while maintaining the conversion effectiveness for low-frequency random movements induced by rotations of the wrist. The impact-based frequency-up conversion technique was experimentally verified by fabricating and testing prototypes of the harvesters tied to the wrist of a person. The obtained results show that the fabricated prototypes can be employed to power autonomous sensor nodes by harvesting electrical energy from nonperiodical or random mechanical movements.

The paper is organized as follows: a mono-axial and bi-axial ball-impact multi-converter piezoelectric harvester description is presented in Section 2. Analytical modelling of a transverse ball impact on a cantilever tip is presented in Section 3. Prototypes and electrical configurations are presented in Section 4. Experimental results are presented in Section 5. Finally, conclusions are drawn in Section 6.

## 2. Mono-Axial and Bi-Axial Ball-Impact Multi-Converter Piezoelectric Harvester Description

**Figure 1:**
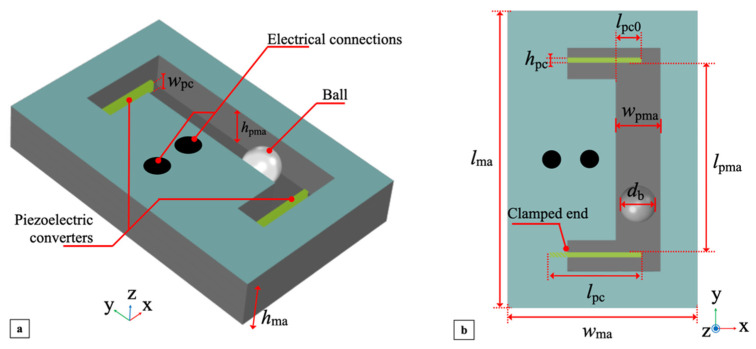
A 3D view (**a**) and top view (**b**) of the mono-axial multi-converter piezoelectric harvester.

The 3D structure of the mono-axial multi-converter piezoelectric harvester is illustrated in Figure 1a. The proposed device adopts two piezoelectric converters (PCs) impacted by a steel ball of diameter *d*_b_ to harvest electrical energy from mechanical energy. The path of the ball is confined within a predefined volume of a rectangular parallelepiped of height *h*_pma_ ≅ 1.2*d*_b_, width *w*_pma_ ≅ 1.2*d*_b_ and length *l*_pma_ ≅ 5.2*d*_b_. The height *h*_pma_, width *w*_pma_ and length *l*_pma_ are defined by the top and bottom layers of the structure, the side walls of the structure and the position of the PCs, respectively.

To achieve mono-axial movements, the dimensions of the device were designed to make the axis along length *l*_pma_, denoted as the y-axis, the impacting axis. Within the delimited volume, the ball can freely move since it is not rigidly or elastically connected to the structure of the harvester.

Therefore, the ball will impact against a single PC when the harvester undergoes suitable external excitation causing the ball to roll and cover the corresponding distance to the PC. Two piezoceramic bimorph elements were employed as the PCs. Each PC is a rectangular parallelepiped of width *w*_pc_, length *l*_pc_ and height *h*_pc_ and was configured as a cantilever by mounting the face *l*_pc_ × *h*_pc_ in the xy-plane, as shown in Figure 1b.

**Figure 2:**
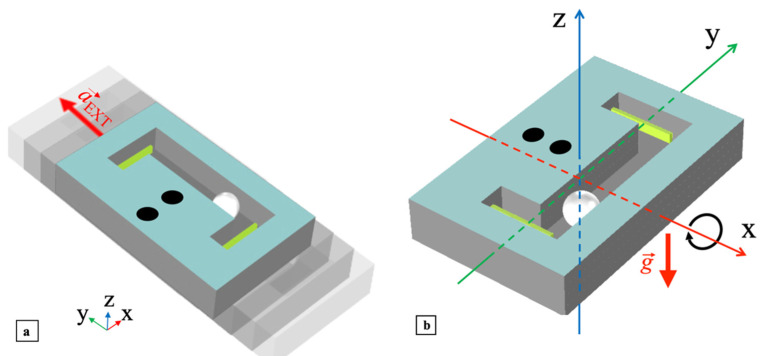
Excitations to which the mono-axial harvester is sensitive: shake applied along the y-axis (**a**) and tilt around the x-axis (**b**).

**Figure 3:**
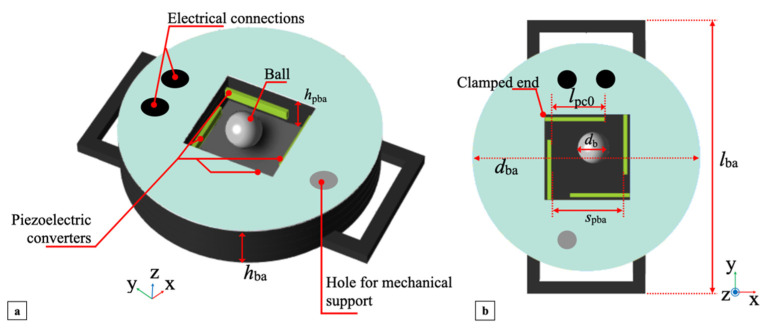
3D view (**a**) and top view (**b**) of the bi-axial multi-converter piezoelectric harvester.

The clamped end of the cantilever was placed at 1/5 of *l*_pc_, while the fraction of the length exposed to impacts is *l*_pc0_ ≅ 0.16*l*_pc_. In particular, *w*_pma_ and *l*_pc0_ were selected to make the impact happen at the cantilever tips to maximize the induced free oscillations. The overall harvester structure is boxed in a rigid frame with width *w*_ma_, length *l*_ma_ and height *h*_ma_ and includes electrical connections to the outputs of the embedded PCs. Assuming for reference that the harvester xy-plane is horizontal, two main different mechanical excitations can produce impacts with a PC, namely a shake applied along the y-axis or a tilt induced around the x-axis, as shown in Figure 2a,b, respectively, plus their combinations. In the first case, the external acceleration ${\vec{a}}_{\mathrm{ext}}$ produced by the shake, will directly cause the ball to impact one of the cantilevers. In the second case, the tilt around the x-axis will move the ball towards one of the cantilevers due to the action of the gravity acceleration $\vec{g}$. The 3D structure of the bi-axial multi-converter piezoelectric harvester is illustrated in Figure 3a. The proposed bi-axial harvester exploits four PCs and a steel ball identical to the mono-axial harvester as the moving element. The path of the ball is confined within a predefined volume of a squared parallelepiped of height *h*_pba_ ≅ 1.2*d*_b_ and width *s*_pba_ ≅ 2.3*d*_b_.

The height is delimited by the top and bottom layers of the structure, while the sides are bounded by the four PCs. The parallelepiped dimensions were designed to make the x and y the impacting axes, therefore implementing a bi-axial ball-impact multi-converter piezoelectric harvester. As in the mono-axial harvester, the ball can move freely within the delimited volume since it is not rigidly or elastically connected to the structure. The ball will impact against a PC when the external excitation, applied to the harvester, causes the ball to move in either direction in the plane until it reaches one boundary.

In addition, subsequent impacts on different PCs can occur due to bouncing and angled deviation of the ball path. Four piezoceramic bimorph elements were employed as the PCs. As in the mono-axial harvester, the piezoelectric converters are rectangular parallelepipeds mounted as cantilevers with the face *l*_pc_ × *h*_pc_ oriented in the xy-plane, as shown in Figure 3b. The clamped end of each cantilever was placed at 1/5 of length *l*_pc_, while the length exposed to impacts is *l*_pc0_ ≅ 0.53*l*_pc_ to keep the ball trajectory in the central region of the confined volume. The harvester structure was assembled in a rigid frame of length *l*_ba_, composed of cylinder height *h*_ba_, and diameter *d*_ba_ with electrical connections to the outputs of the embedded PCs and a hole for the mechanical support of external circuitry.

**Figure 4:**
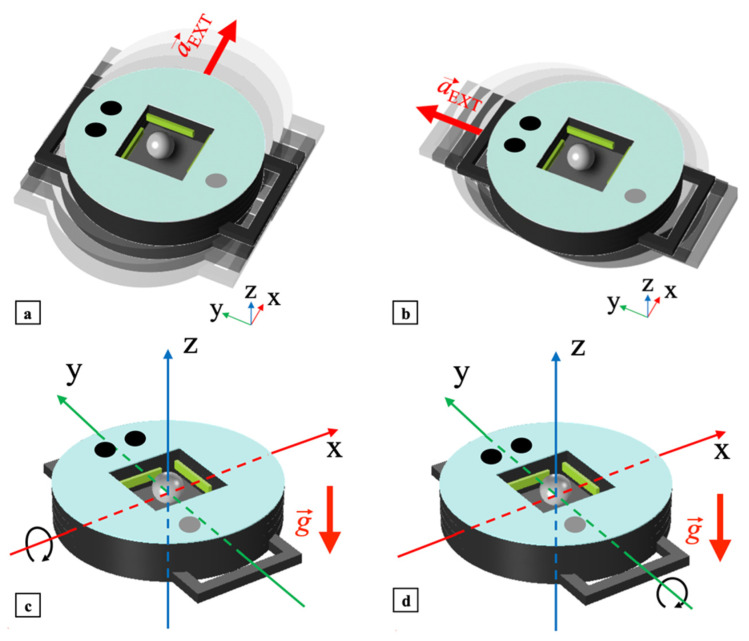
Excitations to which the bi-axial harvester is sensitive: shake applied along the *x*- (**a**) and *y*-axis (**b**) and tilt around the *x*- (**c**) and *y*-axis (**d**).

Assuming for reference that the harvester xy-plane is horizontal, four main different excitations can produce impacts on the PCs, namely a shake applied along the x- or y-axis, a tilt induced around the x- or y-axis, as shown in Figure 4a–d, respectively, plus their combinations. As in the mono-axial harvester, in the case of shake excitations, external acceleration ${\vec{a}}_{\mathrm{ext}}$ will cause the ball to impact the cantilevers, while for tilts gravity acceleration $\vec{g}$ will drive the impacts.

## 3. Analytical Modelling of a Transverse Ball Impact on a Cantilever Tip

**Figure 5:**
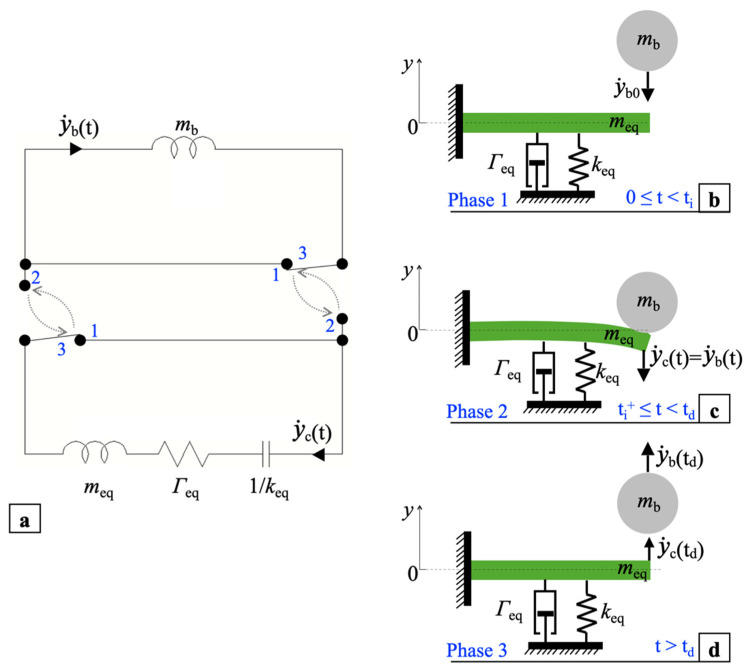
Lumped-element equivalent circuits (**a**) and equivalent simplified mechanical model of a single ball impact piezoelectric converter before the impact (**b**), after the impact (**c**), and after the detachment of the ball from the cantilever (**d**).

The mechanical behavior of an impact happening between the ball and a single cantilever can be described by the simplified one-dimensional mechanical model and equivalent circuits shown in Figure 5. The model is composed of an elastic cantilever representing the piezoelectric converter impacted by a ball that moves towards its tip along the transverse y-axis, i.e., perpendicularly to the cantilever surface. According to the direct electro-mechanical (EM) analogy [51], the mechanical model can be analyzed by employing its equivalent electro-mechanical lumped-element circuit. The inductance, resistance, and capacitance represent in electrical formalism the equivalents of the mass, mechanical resistance, and elastic compliance, respectively. Specifically, the ball is modelled with a rigid mass *m*_b_, while the cantilever with an effective mass-spring-damper system, with equivalent mass *m*_eq_, elastic compliance 1/*k*_eq_ and mechanical resistance *Γ*_eq_, as shown in Figure 5a.

The ball impact on the cantilever is modeled considering three different time intervals named phases 1, 2 and 3 occurring before the impact, just after the impact, and after the ball detaches from the cantilever tip, respectively. The transitions among the three phases were modeled by the commutations of two ideal switches, assumed lossless and without a time delay.

Variables *y*_c_(t) and *y*_b_(t) represent the bending displacement of the cantilever tip and the displacement of the ball with respect to the cantilever equilibrium point *y*_c_ = 0, respectively, while ${\dot{y}}_{c}(t)$ and ${\dot{y}}_{b}(t)$ denote the cantilever tip and ball velocities, respectively.

In phase 1, during the time interval before the impact, 0 ≤ t < t_i_, where t_i_ is the time at which the impact occurs, neglecting the gravitational acceleration and considering the velocities as positive in the direction of the arrows, the ball is assumed in uniform rectilinear motion with constant velocity ${\dot{y}}_{b}(t)={\dot{y}}_{b0}$, as shown in Figure 5b. In this case, since the ball and cantilever are not interacting yet, the switches in Figure 5a are in position 1 thus creating two equivalent lumped-element circuits which are separate and noninteracting. The mass *m*_b_ and, according to the EM analogy, the equivalent inductance *m*_b_ in turn, is considered to have an initial current condition ${\dot{y}}_{b}(0)={\dot{y}}_{b0}$ whereas the cantilever is supposed at rest at its equilibrium point, i.e., ${\dot{y}}_{c}(t)$ and *y*_c_(t) = 0. Therefore, the capacitance 1/*k*_eq_ is initially uncharged and the initial current in the inductance *m*_eq_ is zero.

At time t = t_i_ the ball impacts the tip of the cantilever which bends causing an inelastic collision [52]. In phase 2, during the time interval t_i_^+^ ≤ t < t_d_, between the impact and detachment time t_d_, the ball and the cantilever remain joined moving downwards with the same velocity, i.e., ${\dot{y}}_{b}(t)={\dot{y}}_{c}(t)={\dot{y}}_{b}({t_{i}}^{+})$, as shown in Figure 5c.

In this case a coupled system is created in which the overall spring constant and the mechanical resistance are those of the cantilever and the overall equivalent mass is the sum of the masses *m*_eq_ and *m*_b_. Accordingly, the switches in Figure 5a are in position 2 and, in the resulting equivalent circuit, the two inductors are in series. In this time interval, the ball and the cantilever tip initially move downwards until velocity ${\dot{y}}_{c}(t)$ nulls and displacement *y*_c_ reaches the negative maximum. Then the cantilever tip and the ball move upward until their maximum velocity, i.e., ${\dot{y}}_{b}(t_{d})={\dot{y}}_{c}(t_{d})={\dot{y}}_{b}(t_{d})$, is reached at *y*_c_ = 0.

Once reached such condition, in phase 3 for t > t_d_ the ball detaches from the cantilever, as shown in Figure 5d. The ball leaves the cantilever with uniform rectilinear motion with velocity ${\dot{y}}_{b}(t)={\dot{y}}_{b}(t_{d})$ while the cantilever, due to its inertia, undergoes free oscillations until the mechanical damper dissipates all the energy transferred by the ball. The ball and cantilever return to behave as independent systems, the switches in Figure 5a are in position 3, coincident with position 1, and, correspondingly, the equivalent circuits are again uncoupled.

The second-order differential equation and initial conditions for the cantilever after the ball detachment for t > t_d_ are:

(1) $$\{\begin{array}{l} m_{\mathrm{eq}}{\ddot{y}}_{c}(t-t_{d})+\Gamma_{\mathrm{eq}}{\dot{y}}_{c}(t-t_{d})+k_{\mathrm{eq}}y_{c}(t-t_{d})=0\\ {\dot{y}}_{c}(t_{d})=\dot{y}(t_{d})\\ y_{c}(t_{d})=0 \end{array}\;t>t_{d}$$

The solution of Equation (1) is the displacement *y*_c_(t), resulting in:

(2) $$y_{c}(t)=\frac{\dot{y}(t_{d})}{2\pi f_{\mathrm{dr}}}\sin(2\pi f_{\mathrm{dr}}(t-t_{d}))e^{{-(t-t}_{d})/\tau },$$

where *f*_dr_ is the damped resonant frequency given by:

(3) $$f_{\mathrm{dr}}=\sqrt{f_{r}^{\;2}-\frac{1}{{\left(2\pi \tau \right)}^{2}}}$$

and

(4) $$f_{r}=\frac{1}{2\pi }\sqrt{\frac{k_{\mathrm{eq}}}{m_{\mathrm{eq}}}},$$

(5) $$\tau =\frac{2m_{\mathrm{eq}}}{\Gamma_{\mathrm{eq}}}$$

are resonant frequency *f*_r_ and decay time *τ* of the cantilever, respectively.

From Equation (2), the velocity of the cantilever tip ${\dot{y}}_{c}$(t) can be derived as follows:

(6) $${\dot{y}}_{c}(t)=\left[-\frac{1}{\tau \;{2\pi f}_{\mathrm{dr}}}\;{\sin(2\pi f}_{\mathrm{dr}}{(t-\mathrm{td}))+\cos(2\pi f}_{\mathrm{dr}}\left(t-\mathrm{td})\right)\right]\dot{y}(t_{d})e^{{-(t-t}_{d})/\tau }.$$

**Figure 6:**
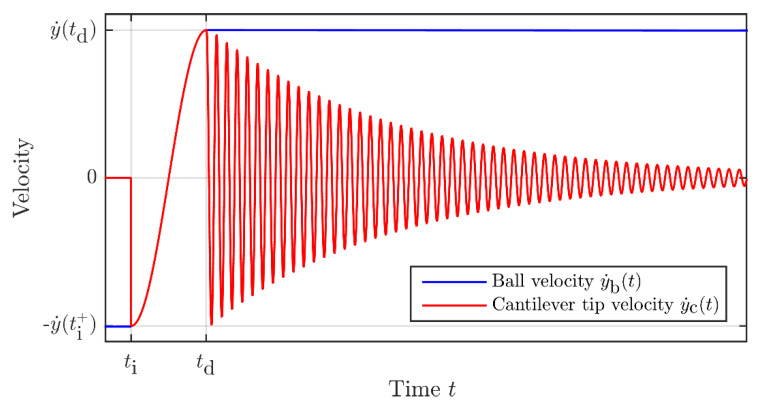
Typical behaviors of the velocity of the ball, ${\dot{y}}_{b}(t)$ (blue curve), and of the cantilever tip, ${\dot{y}}_{c}(t)$ (red curve), caused by an impact.

The typical behaviors of the velocity of the ball, ${\dot{y}}_{b}(t)$ and of the cantilever tip ${\dot{y}}_{c}(t)$ derived from the model and the resulting equations, are illustrated in Figure 6 with blue and red plots, respectively.

## 4. Prototypes and Electrical Configurations

Prototypes of the mono-axial and bi-axial ball-impact multi-converter harvesters described in Section 2 were fabricated employing 1.6 mm-thick FR-4 sheets as structural elements in a multi-layer arrangement.

**Figure 7:**
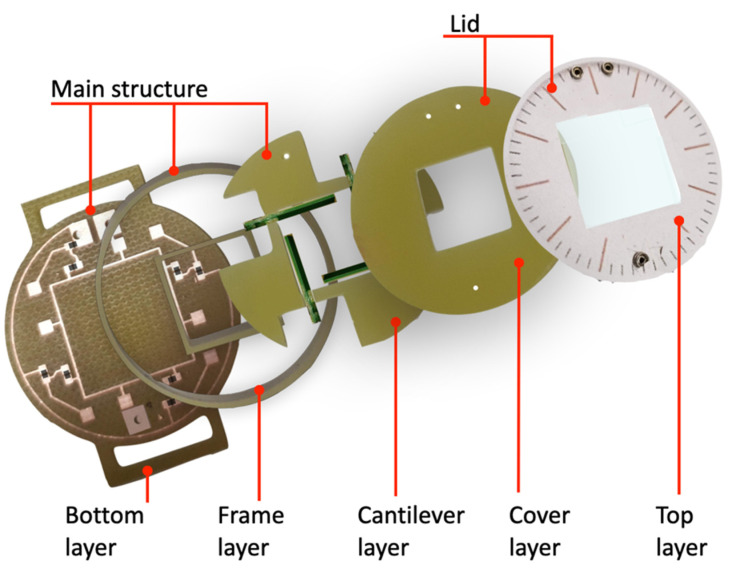
Images of the FR-4 layers that compose the main structure and the lid of the fabricated bi-axial multi-converter harvester prototype.

Figure 7 shows the five processed FR-4 layers employed in the bi-axial harvester. A similar composite structure was developed for the mono-axial harvester. The bottom layer embeds the printed circuit board (PCB) and the rectifier circuits of each PC. The frame layer is placed on the bottom layer and is used to protect the electrical components and the PCB and to support the cantilever layer, where each PC was glued firmly into the clamped end. As per the piezoelectric converters, the three FR-4 layers, which compose the main structure of the harvester, have also been glued together with an epoxy resin.

To easily access the steel ball placed inside the harvester, a lid was built by employing a cover layer and a top layer glued together. Furthermore, a transparent plastic film and a paper stamp were glued on the top layer. The bi-axial harvester was embedded with three key pins, which provide mechanical support and correct alignment between the lid and structure body. Two of the three pins were also employed as electrical connections to electrically connect additional external circuitry to the harvester.

**Figure 8:**
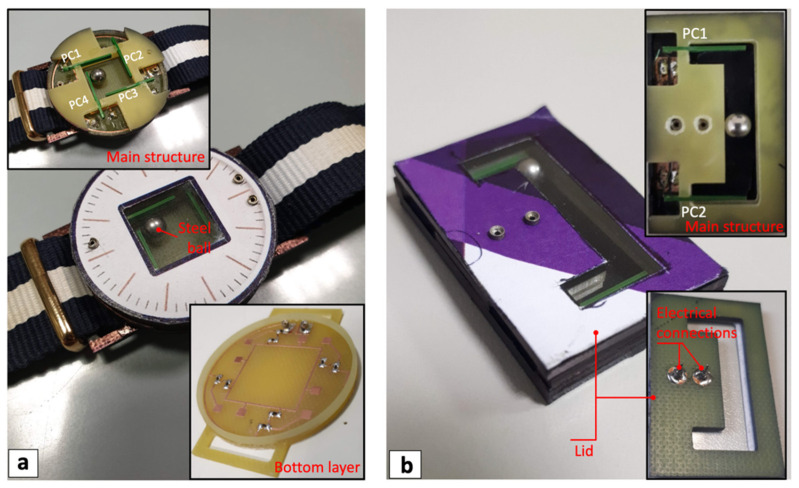
Images of the mono-axial (**a**) and bi-axial (**b**) multi-converter harvester prototypes with views of the main structures, bottom layer, and lid.

Images of the bi-axial harvester prototype are shown in Figure 8a, with views of the main structure and the bottom layer that compose the harvester. In particular, it is possible to notice the four piezoelectric converters, PC1–PC4, in the main structure, and the soldered electrical components in the bottom layer. Images of the mono-axial prototype are shown in Figure 8b with views of the main structure and the lid that compose the harvester. The two piezoelectric converters, PC1 and PC2, in the main structure and the electrical connections in the lid can be seen.

Commercial piezoceramic bimorph elements (RS-pro 285–784) were adopted as PCs capable of converting mechanical to electrical energy.

**Figure 9:**
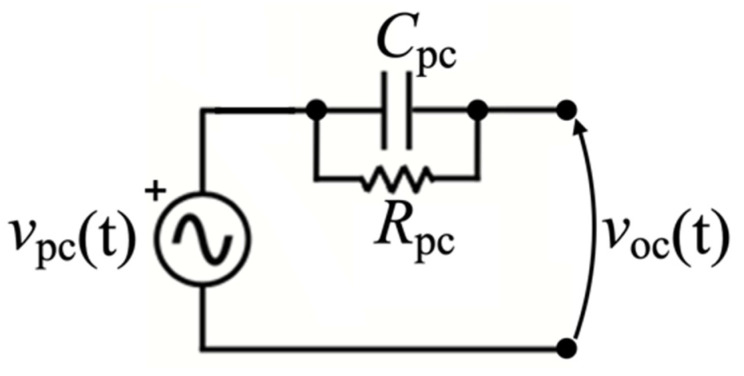
Thévenin equivalent model of a single PC.

A single PC can be represented by its Thévenin equivalent circuit, comprising a voltage source, *v*_pc_(t), in series with the internal impedance of the piezoelectric element [53], made by the parallel of capacitance *C*_pc_ and resistance *R*_pc_, as shown in Figure 9.

The electrical components *C*_pc_ and *R*_pc_ can be assumed to be frequency-independent, while *v*_pc_(t) determines open-circuit voltage *v*_oc_(t) and reflects the dynamic mechanical response of the cantilever for each impact event, described in Section 3 and reported in Figure 6. Physical properties of the PCs and steel ball with the geometrical dimensions of the developed mono-axial and bi-axial harvesters are listed in Table 1. Piezoceramic elements can suffer possible long-term durability limitations after repeated impacts due to the intrinsic brittleness and sensitivity to the phenomenon of fatigue. The mass (0.51 g) and diameter (5 mm) of the ball were properly selected to keep mechanical properties compatible with wrist-worn applications and to prevent damage to the PCs. As a different approach, to further avoid overstress to PCs, solutions based on indirect impacts, e.g., impacts happening on the substrate on which the piezoelectric transducer is installed, or magnetic interactions to trigger the frequency-up conversion can be exploited [17,54].

**Figure 10:**
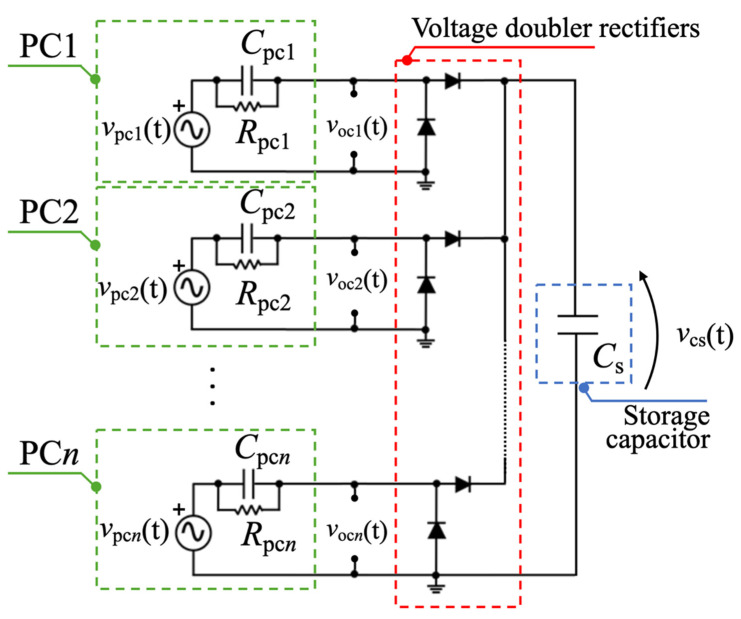
Passive parallel-like electrical configuration of a generic n-multi-converter piezoelectric harvester.

To power the electronics within a sensor module, AC voltages *v*_pc*n*_(t), where *n* = 1, 2 for the mono-axial harvester and *n* = 1–4 for the bi-axial harvester, provided by each PCs had to be rectified [46,55]. Voltage-doubler rectifiers based on BAS116LP3-7 diodes were connected in parallel [56] to charge a single storage capacitor *C*_s_, as reported in Figure 10. The voltage-doubler rectifiers were soldered on the bottom layer of each harvester while the storage capacitor was connected externally to the harvester through electrical connections. The proposed circuit was designed for PCs excited by discontinuous vibrations with an irregular intensity and repetition rate. To preserve the amount of charge extracted and stored in the capacitor *C*_s_ when no excitation is present, leakage currents were minimized for all selected components.

## 5. Experimental Results

**Figure 11:**
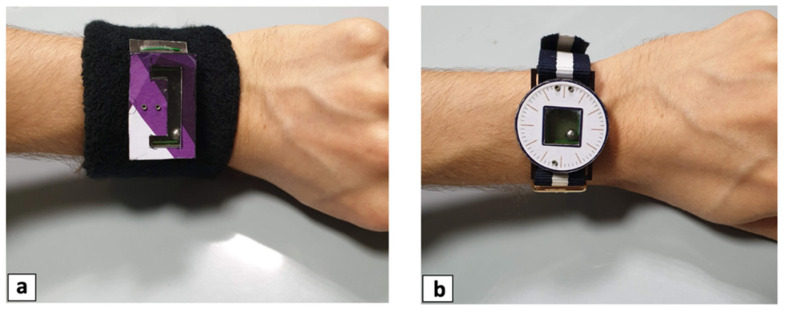
Images of the wearable mono-axial (**a**) and bi-axial (**b**) multi-converter harvester prototypes.

The developed mono-axial and bi-axial prototypes were experimentally tested to validate the proposed ball-impact multi-converter mechanism and to estimate the performances in terms of measured open-circuit voltage and harvested electrical energy. The harvesters were provided with wristband and watchband to easily tie the prototypes to the wrist of a person, as illustrated in Figure 11a,b.

The open-circuit voltages, *v*_ocn_(t), of the PCs that compose the harvesters were first acquired employing a MSOX3014A mixed signal oscilloscope, bypassing the diode-based voltage-doubler rectifiers. Taking into account the load effect of the input impedance of the oscilloscope, composed of the parallel of capacitor *C*_load_ = 14 pF and resistor *R*_load_ = 1 MΩ, the measured open-circuit voltage of *n-*PC *v*_ocM*n*_(*f*) in the frequency domain results:

(7) $$v_{\mathrm{ocMn}}{(f\;)=v}_{\mathrm{ocn}}\left(f\;\right)\frac{R_{\mathrm{pc}}R_{\mathrm{load}}\;}{R_{\mathrm{pc}}{+R}_{\mathrm{load}}}\frac{{1+j2\pi f\;R}_{\mathrm{pc}}C_{\mathrm{pc}}}{1+j2\pi f\;\frac{R_{\mathrm{pc}}R_{\mathrm{load}}\;}{R_{\mathrm{pc}}{+R}_{\mathrm{load}}}\left(C_{\mathrm{pc}}{+C}_{\mathrm{load}}\right)},$$

Considering this, as reported in Table 1, *R*_load_ = *R*_pc_, Equation (7) becomes:

(8) $$v_{\mathrm{ocMn}}{(f\;)=v}_{\mathrm{ocn}}\left(f\;\right)\frac{1}{2}\frac{{1+j2\pi f\;R}_{\mathrm{pc}}C_{\mathrm{pc}}}{1+j2\pi f\;\frac{R_{\mathrm{pc}}}{2}\left(C_{\mathrm{pc}}{+C}_{\mathrm{load}}\right)}.$$

**Figure 12:**
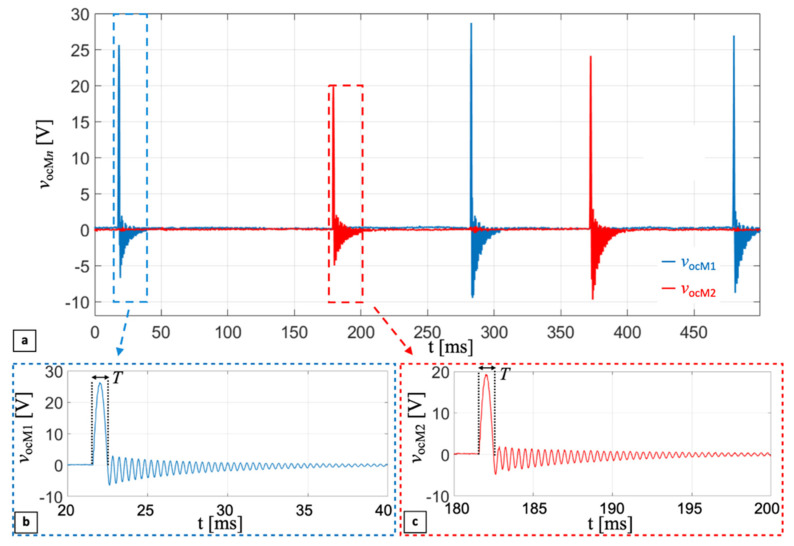
Measured open-circuit voltages, *v*_ocM*n*_(t), of piezoelectric converter PC1 (blue curve) and PC2 (red curve) of the mono-axial harvester (**a**). Enlarged images of a single impact happening against PC1 (**b**) and PC2 (**c**).

Figure 12a reports the measured open-circuit voltages, *v*_ocM1_(t) (blue curve), and *v*_ocM2_(t) (red curve), of PC1 and PC2 for the mono-axial harvester, as a function of time, respectively. The five subsequent impacts were induced by rotations of the wrist, at an excitation rate of about 4 Hz. Figure 12b,c show enlarged images of a single impact happening on PC1 and PC2, respectively. The enlarged plots show an impact interval *T* of approximately half a cycle of a sine wave, whose peak value is related to the mass of the ball, the relative velocity between the cantilever and the ball at impact, and therefore to the excitation acceleration.

The following damped sinusoid is due to the cantilever undergoing free oscillations after the ball detachment, lasting until the mechanical damper dissipates all the energy transferred to the system by the ball, as described in Section 3. Frequency *f*_dr_ of the damped sinusoidal oscillation is around 2.7 kHz. The reported experimental results show the frequency-up conversion of the impact technique. Low-frequency movements, induced by rotations of the wrist, thanks to the impact-based technique, make the PCs provide AC voltage at a frequency almost three orders of magnitude higher than the excitation frequency.

Considering the measured open-circuit voltages, *v*_ocM*n*_(t), reported in Figure 12, and the trade-off between stored electric energy and charging time of a capacitor, an external storage capacitor *C*_s_ = 220 nF was connected to the electrical connections of the mono-axial harvester, including the diode-based voltage-doubler rectifiers, as described in Section 4. Voltage *v*_cs_(t) on the capacitor was acquired employing a Keithley 6517A electrometer used as a voltage buffer with an input impedance equivalent to capacitor *C*_el_ = 20 pF in parallel with a resistor *R*_el_ > 200 TΩ.

**Figure 13:**
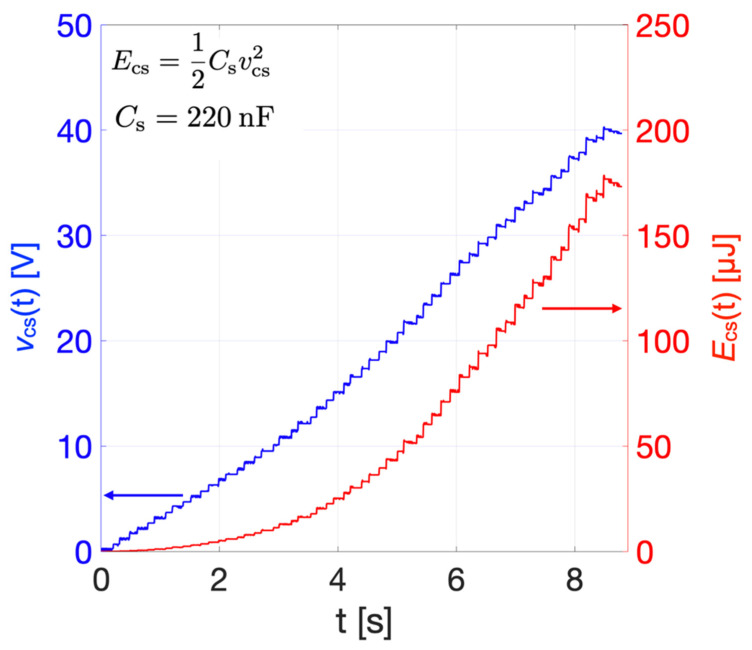
Measured rectified voltage *v*_cs_(t) (blue curve) and estimated energy *E*_cs_ (red curve) stored in capacitor *C*_s_, induced by multiple consecutive impacts.

Figure 13 shows the *v*_cs_(t) (blue curve) produced by multiple consecutive impacts induced by repetitive wrist rotations. Energy *E*_cs_ stored in the capacitor, plotted as the red curve, was derived as follows:

(9) $$E_{\mathrm{cs}}(t)=\frac{1}{2}C_{s}v_{\mathrm{cs}}(t{)}^{2}.$$

After 8.5 s of consecutive impacts, *v*_cs_(t) reached 40.2 V, which, from Equation (9), corresponds to a stored energy of 178 μJ.

**Figure 14:**
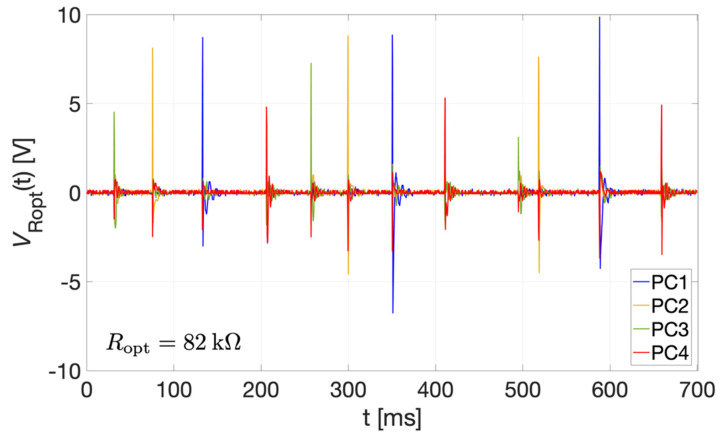
Measured voltages *v*_Ropt_(t) as a function of time with three different impacts happening against each PCs of the bi-axial harvester PC1, PC2, PC3, and PC4, reported with blue, yellow, green, and red curves, respectively.

To measure the electrical power provided by the bi-axial harvester, each PCs was directly connected to a resistor *R*_opt_ of 82 kΩ without the rectifier circuit. Such resistance value was chosen to match the magnitude of the cantilever impedance at the corresponding resonant frequency *f*_r_ according to the data reported in Table 1. Voltages *v*_Ropt_(t) of each *R*_opt_ connected to each PC were acquired for the bi-axial harvester. Figure 14 reports the measured voltages *v*_Ropt1_(t) (blue curve), *v*_Ropt2_(t) (yellow curve), *v*_Ropt3_(t) (green curve) and *v*_Ropt4_(t) (red curve), for PC1, PC2, PC3 and PC4, respectively, as a function of time. Three different impacts were induced on each PC by means of wrist rotations.

Based on the experimental results reported in Figure 14, considering the instantaneous power *P*_Ropt_(t) from each PC, given by:

*P*_Ropt_(t) = *v*^2^_Ropt_(t)/*R*_opt_ (10)

**Figure 15:**
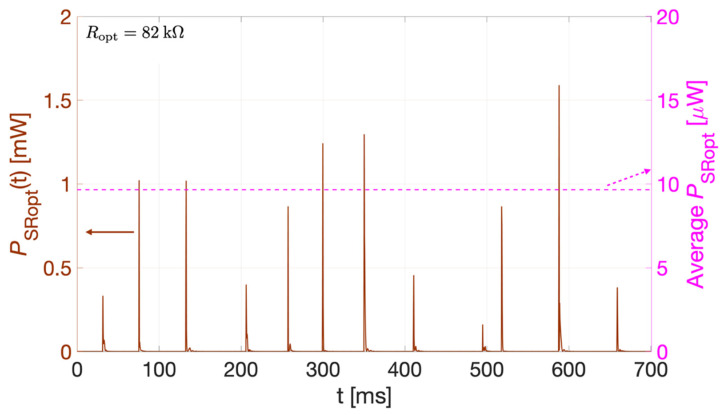
Sum of the instantaneous power *P*_SRopt_(t) (brown curve) and its average value (pink dotted line), obtained from the experimental results reported in Figure 14.

Figure 15 shows the sum of the instantaneous powers from all PCs, *P*_SRopt_(t) (brown curve), and its average value (pink dotted line). The maximum peak value of *P*_SRopt_(t) is 1.58 mW while the average over 0.7 s results 9.65 μW. To evaluate the performances of the bi-axial harvester in terms of energy stored and charging time, two different values of storage capacitors *C*_s_ = 220 nF and *C*_s_ = 1 μF were tested. The external storage capacitor was connected to the electrical connections of the bi-axial harvester, including the diode-based voltage-doubler rectifier circuit, as described in Section 4. As per the mono-axial harvester, the voltage across the capacitor, *v*_cs_(t), was acquired employing the electrometer.

**Figure 16:**
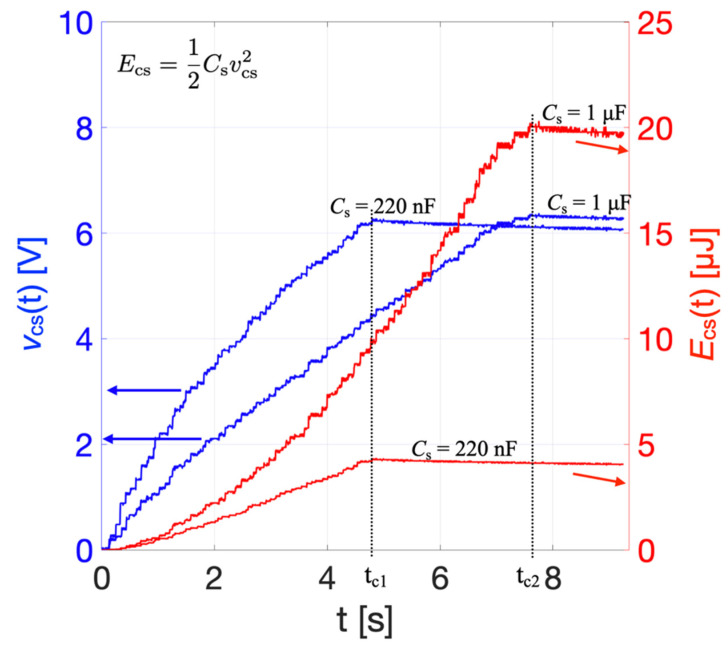
Measured rectified voltage *v*_cs_(t) (blue curves) and estimated electrical energy *E*_cs_(t) (red curves) stored employing two different values of storage capacitors C_s_.

Figure 16 shows the measured rectified voltages, *v*_cs_(t) (blue curves), across the capacitor *C*_s_ produced by multiple consecutive impacts induced by repetitive rotations of the wrist. The corresponding electrical energies, *E*_cs_, stored in the capacitor obtained from Equation (9) are also plotted (red curves).

To compare the effects that different values of storage capacitor *C*_s_ have on the charging process, the bi-axial harvester was subjected to consecutive controlled impacts until the voltage *v*_cs_(t) reached the preset threshold value of 6.2 V. Employing *C*_s_ = 220 nF, a charging time t_c1_ = 4.8 s was required to reach the threshold voltage, which was shorter than in the case with *C*_s_ = 1 μF where the charging time was t_c2_ = 7.6 s. However, for *C*_s_ = 220 nF the energy stored was almost one fourth of the energy stored with *C*_s_ = 1 μF. As expected, increasing the value of the storage capacitance at parity of threshold voltage increases both the stored energy and the charging time, which requires a trade-off in the selection of *C*_s_.

## 6. Conclusions

In this work, mono-axial and bi-axial ball-impact piezoelectric harvesters are presented. The proposed harvesters exploit multiple piezoelectric converters (PCs) configured as cantilever to harvest electrical energy from mechanical energy employing a steel ball as a free-to-move element impacting the PCs.

The impact between the ball and the PCs happens as soon as the external excitation applied to the harvester by a shake or a tilt causes the ball to roll towards a cantilever. The path of the ball is confined within a predefined volume, designed to make the overall size compliant with the dimension constraints of wearable and unobtrusive devices.

A detailed description of the design of the harvesters is reported along with a basic electro-mechanical modeling of the impact between the ball and a single PC configured as a cantilever. Prototypes of the mono-axial and bi-axial ball-impact multi-converter harvesters were developed and experimentally tested by tying them to the wrist of a person by means of a wristband and watchband. Applying excitation frequencies of about 4 Hz by means of wrist rotations, the open-circuit voltages of the PCs of the mono-axial harvester and the voltages of the PCs connected to an optimal resistive load for the bi-axial harvester were measured. As theoretically predicted from the model, the measured trends exhibit a distinctive shape at impact, followed by a post-impact damped sinusoidal oscillation of around 2.7 kHz, which is related to the resonant frequency of the clamped unloaded cantilever.

The reported experimental results validate the frequency-up conversion of the impact technique. AC voltages with a frequency of almost three orders of magnitude higher than the excitation frequency were measured. The maximum peak value of the sum of the instantaneous powers for optimal resistive load resulted in 1.58 mW, while the average value over 0.7 s was 9.65 μW. The performances of the harvesters, in terms of stored energy and charging time, were evaluated connecting a storage capacitor, *C*_s_ = 220 nF, through the electrical terminals including passive diode-based rectifier circuits connected in parallel. After 8.5 s of consecutive impacts induced by wrist rotations, a voltage, *v*_cs_(t), of 40.2 V across *C*_s_ was obtained, which corresponds to a stored energy of 178 μJ. The proposed harvesters are suitable for sensor nodes where electrical energy has to be scavenged from low-frequency nonperiodical or random mechanical movements, such as human motion.

Ongoing activities are considering reliability and fatigue issues and are analyzing the effect of impacts occurring in different points of the harvester structure.

## Figures and Tables

**Table 1:** Physical properties of the employed PCs and steel ball and geometrical dimensions of the developed mono-axial and bi-axial harvesters.

Description	Parameter	Value
**Piezoelectric Converter (PC)**		
width	*w*_pc_	1.5 mm
height	*h*_pc_	0.6 mm
length	*l*_pc_	15 mm
capacitance	*C*_pc_	750 ± 170 pF
resistance	*R*_pc_	1 MΩ
		
**Steel Ball**		
diameter	*d*_b_	5 mm
mass	*m*_b_	0.51 g
		
**Mono-Axial Harvester**		
length	*l_ma_*	40 mm
width	*w*_ma_	25.5 mm
height	*h*_ma_	8 mm
parallelepiped length	*l*_pma_	25.8 mm
parallelepiped width	*w*_pma_	6 mm
parallelepiped height	*h*_pma_	6.4 mm
PC length exposed to impact	*l*_pc0_	2.5 mm
		
**Bi-Axial Harvester**		
length	*l_ba_*	44 mm
height	*h_ba_*	8 mm
diameter	*d_ba_*	36 mm
parallelepiped side	*s*_pba_	11.6 mm
parallelepiped height	*h*_pba_	6.4 mm
PC length exposed to impact	*l*_pc0_	8.05 mm
